# Boosting the local anodic oxidation of silicon through carbon nanofiber atomic force microscopy probes

**DOI:** 10.3762/bjnano.6.20

**Published:** 2015-01-19

**Authors:** Gemma Rius, Matteo Lorenzoni, Soichiro Matsui, Masaki Tanemura, Francesc Perez-Murano

**Affiliations:** 1 Nagoya Institute of Technology, NITech, Gokiso, Showa, 466-8555 Nagoya, Japan; 2 Institut de Microelectronica de Barcelona, IMB-CNM-CSIC, Campus UAB 08193 Bellaterra, Spain

**Keywords:** carbon nanofiber, dynamic mode, local anodic oxidation, nanopatterning

## Abstract

Many nanofabrication methods based on scanning probe microscopy have been developed during the last decades. Local anodic oxidation (LAO) is one of such methods: Upon application of an electric field between tip and surface under ambient conditions, oxide patterning with nanometer-scale resolution can be performed with good control of dimensions and placement. LAO through the non-contact mode of atomic force microscopy (AFM) has proven to yield a better resolution and tip preservation than the contact mode and it can be effectively performed in the dynamic mode of AFM. The tip plays a crucial role for the LAO-AFM, because it regulates the minimum feature size and the electric field. For instance, the feasibility of carbon nanotube (CNT)-functionalized tips showed great promise for LAO-AFM, yet, the fabrication of CNT tips presents difficulties. Here, we explore the use of a carbon nanofiber (CNF) as the tip apex of AFM probes for the application of LAO on silicon substrates in the AFM amplitude modulation dynamic mode of operation. We show the good performance of CNF-AFM probes in terms of resolution and reproducibility, as well as demonstration that the CNF apex provides enhanced conditions in terms of field-induced, chemical process efficiency.

## Introduction

Scanning probe lithography (SPL) is increasing its relevance among currently employed methods towards miniaturization and investigations at the nanometer scale. Interest of developing SPL-based nanofabrication methods relies on its extraordinary performance in terms of resolution and flexibility, as well as its potential for applications, e.g., in materials/surface science, quantum devices and nanoelectronics [1]. Moreover, SPL has the additional capability of in situ inspection, which provides additional control over the fabrication process including pattern placement [2].

SPL can be performed in a wide variety of instrument configurations and operation modes, such as in scanning tunneling microscopy (STM) or atomic force microscope (AFM). Based on the latter, AFM, it excels in versatility, as its working principle allows AFM to be applied conveniently onto any surface and in a variety of atmospheres [2–4]. Nonetheless, SPL based on AFM can rely on a number of tip–surface interactions (chemical, electrical, thermal, etc.), including tip–sample direct mechanical contact or long range interactions, such as based on van der Waals or electrostatic forces. Because of this, AFM-based SPL can be achieved through oxidation, indentation, as well as various other implementations such as dip-pen nanolithography [5]. Early works on AFM-based SPL logically concerned silicon, as it is the ubiquitous material of modern electronics [6–7]. The application of an electric field between a conductive tip and a silicon substrate under ambient conditions can generate the local anodic oxidation (LAO) of the silicon surface very precisely; intrinsic silicon oxide (SiO*_x_*) patterns are in the single/double-digit nanometer-range [8].

The principle of LAO-AFM is the following: A water meniscus is formed in humid air when the tip comes to close proximity to the surface due to capillary condensation. The formation of the water meniscus can be triggered in non-contact mode by the application of a certain bias voltage between the tip and sample. The water meniscus bridges electrical conduction and provides the anions to enable the chemical reaction. Conditions for oxidation require that hydroxy anions are driven towards the substrate, i.e., the sample should be positively biased [9]. Typical anodic currents are of the order of nanoamperes [10] and their efficiency depends on various conditions, which concern the tip, (e.g., conductance and shape) the tip–sample interplay, (e.g., distance and wetting), and other factors such as sample surface texture or wetting. All those parameters also affect the actual resolution of the LAO-AFM features and process reliability.

The understanding of the conditions for LAO-AFM as well as of the resolution capabilities have been addressed from several viewpoints. Particularly, non-contact LAO-AFM has proven to yield a better resolution and tip preservation than the contact mode [11–12]. The improvement of the water meniscus comes from the control of the water meniscus dimensions, which depends on several parameters, including tip–sample distance, humidity and electrical field. Remarkably, LAO-AFM can be performed in dynamic mode AFM, so it is fully compatible with the standard imaging conditions [7].

One direction for further optimization of LAO-AFM is tip engineering. Tip shape and sharpness plays a crucial role for the LAO-AFM as a main regulator of minimum feature size and electric field [13]. Reversely, when the tip morphologically and chemically degrades during its use, the conditions and the results of LAO-AFM are dramatically affected or even lost. It has been proposed, as one possibility to overcome this issue, the use of carbon nanotube (CNT)-functionalized tips [14–15]. With excellent electronic conduction, mechanical and chemical properties, intrinsic very high aspect ratios and tiny tip radii, CNTs looked very promising for LAO-AFM application. Indeed, both single and multi-walled CNTs showed remarkable patterning capabilities [16]. However, this approach has been nearly abandoned, due to the high cost and poor control upon making CNT probes, as well as characteristic tip-to-tip differences, such as length, diameter, and operational complications, such as CNT buckling [15–16].

In this work, we explore the use of a carbon nanofiber (CNF) as the tip apex of AFM probes for the application of LAO-AFM on silicon substrates in amplitude modulation dynamic mode of operation. In spite of the morphological and chemical resemblance, CNFs and CNTs exhibit fundamental structural differences. Both CNF and CNT are high aspect ratio morphologies (one-dimensional) made primarily of atomic carbon. However, a CNF consists of solid amorphous carbon, while a CNT is a tubular crystalline nanomaterial, therefore we expect both common and distinctive features of CNF as a tool for LAO-AFM, as compared to CNT probes. To the best of our knowledge, we report for the first time the use of CNF for SPL. Our CNFs are batch grown by ion-irradiation upon commercial AFM silicon probes [17–18]. We do not only show the good performance of CNF-AFM probes for LAO-AFM in terms of resolution and reproducibility, but our experimental results demonstrate that CNF apex provides enhanced conditions in terms of field-induced chemical process efficiency.

## Experimental

**Figure 1:**
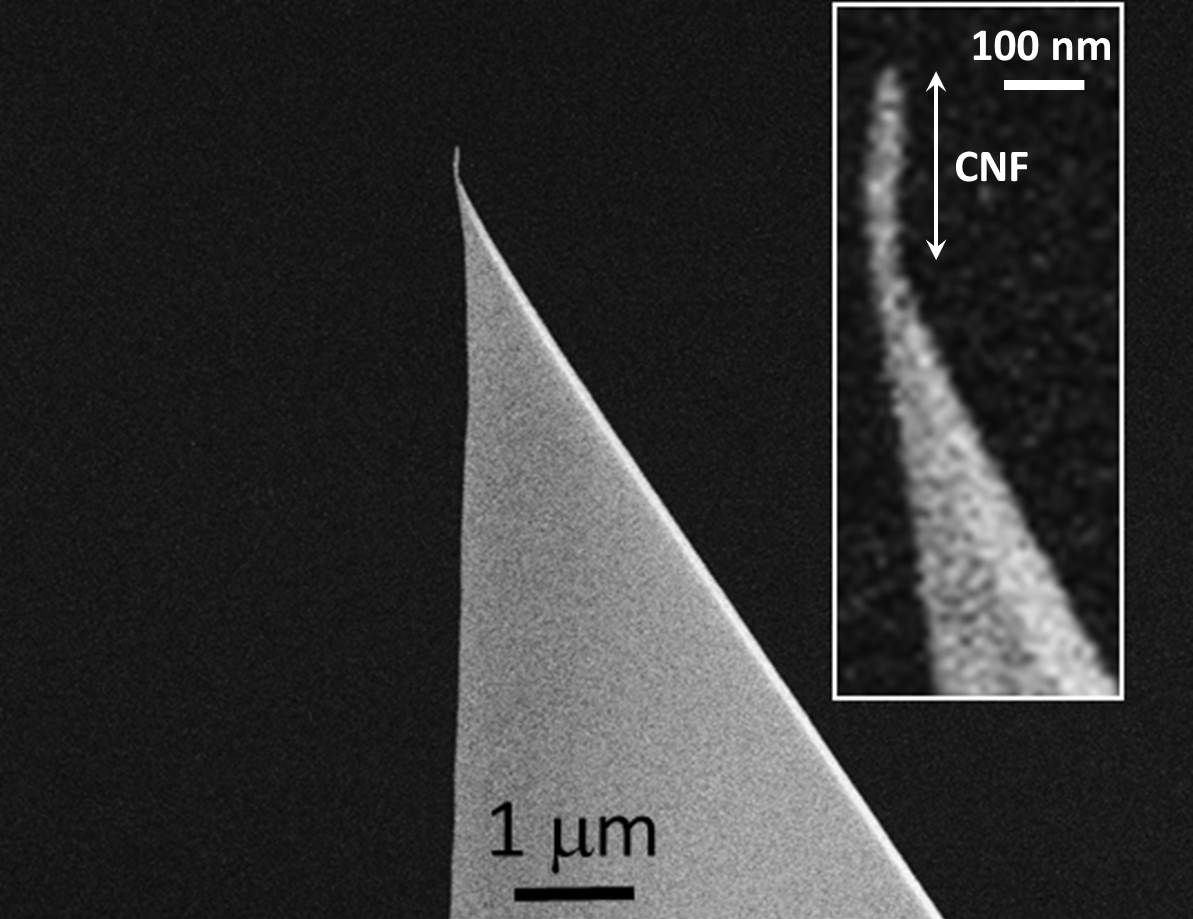
SEM image of a typical example of an as-deposited CNF onto the tip apex of the Si AFM probe.

CNFs are grown on arrays of commercially available AFM cantilevers, non-blade tetragonal-type Si tips made by Olympus (force constant, *k* = 40 N·m^−1^), coated with a thin carbon layer; on typically 3–9 chips per batch [17]. The synthesis is performed in a Kaufman-type ion gun, whose beam diameter is 3 cm, at nearly room temperature. The basal and working pressures are 1.5 × 10^−5^ Pa and 2 × 10^−2^ Pa, respectively. The ion beam energy is 600 eV, and the growth duration is 8 min. CNF elliptical cross section is smaller than 50 nm in diameter, and it is systematically and conveniently aligned with respect to the conical probe as seen in Figure 1. A set of 8 CNFs, which had comparable morphological characteristics have been used for the experiments presented below. The irradiation with Ar^+^ ions is the main factor to induce CNF growth, as described in detail elsewhere [18]. The arrangement of ion-sputtered atomic carbon in the apex of the silicon tip as a CNF results from the re-deposition of ejected carbon atoms, which have diffused along the surface of the Si conical tip. As-grown CNF morphology is characterized by FE-SEM (Hitachi S-4700 operated at 20 keV) and checked (occasionally) at different stages of the LAO-AFM tests (Zeiss, LEO 1530 operated at 3 keV). For comparison purposes, commercially available non-coated doped Si AFM probes from Nanosensors, OTESPA (force constant, *k* = 40 N·m^−1^), are employed.

LAO-AFM and AFM imaging are performed in the amplitude modulation dynamic mode while using relatively stiff cantilevers as specified above. The routines and conditions to perform LAO-AFM in the dynamic mode have been described in [1,19]. In brief, a target location onto the Si substrate is inspected for surface cleanliness. Then, the cantilever free oscillation is set to a low amplitude value (<20 nm), to ensure a close tip–surface distance and the set point amplitude is routinely set to 80% of the free amplitude for imaging when the feedback is active. Under these conditions, attractive forces dominate and, in consequence, it can be inferred that the AFM is operated in non-contact mode. In current experiments we focus on the definition of line patterns. Prior to patterning, the AFM control feedback is disabled and the required voltage is applied. However, in order to keep a constant tip–surface distance, previously the surface inclination with respect to the X–Y piezo-scanning plane is captured and subtracted. All tests are performed at room conditions, with a temperature of 25 °C and under a controlled relative humidity ranging from 20 to 40%. The Si substrates consist of chips cut from Si(100) wafers. Organic contamination on the chips was removed by oxygen plasma etching before the measurements. The native oxide has not been removed.

## Results

### Kinetics of CNF-LAO-AFM

**Figure 2:**
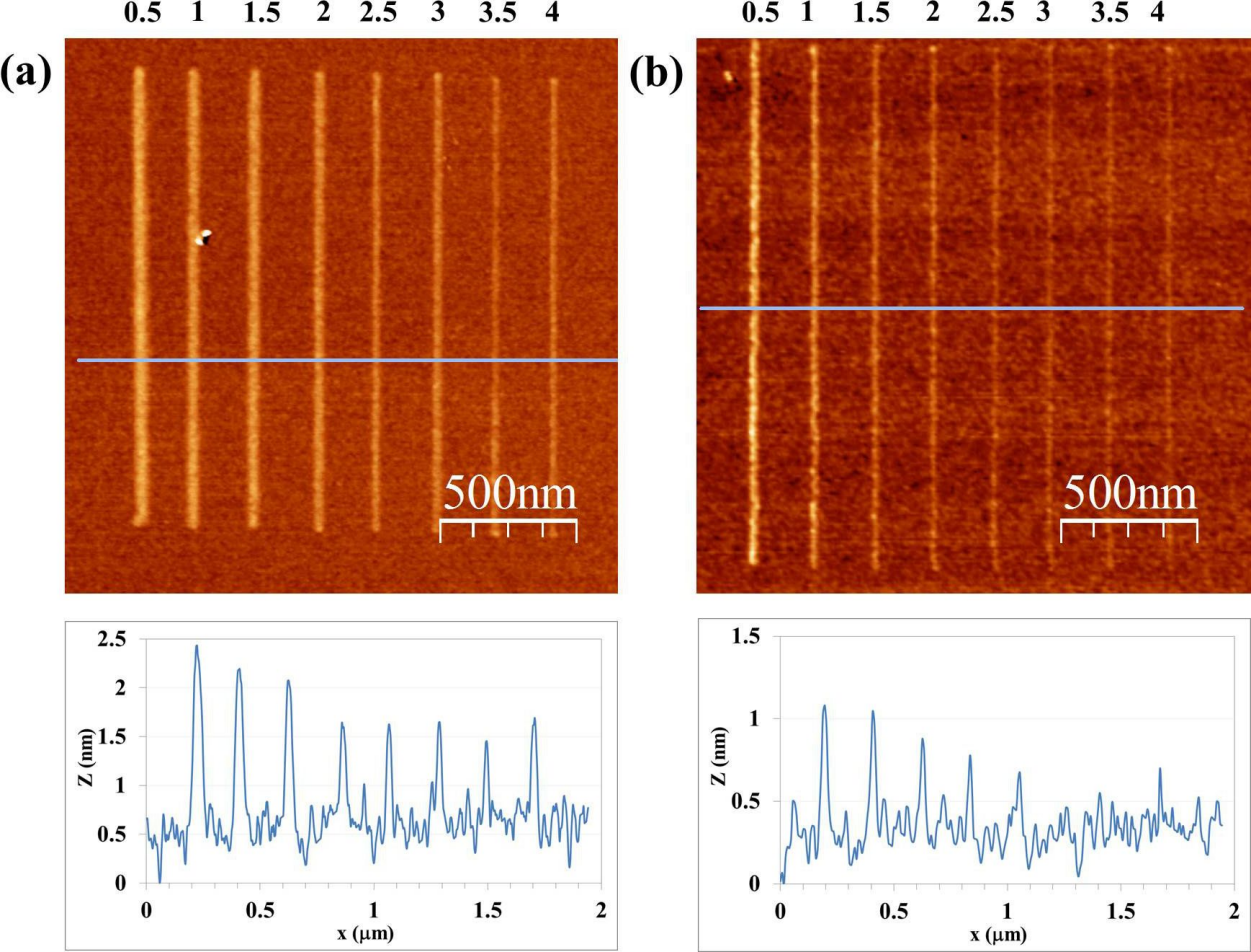
Results of LAO-AFM on Si with CNF (a) and Si (b) probes as a function of the writing speed (μm/s). Bias voltage is 20 V, relative humidity (RH) is 36%.

Figure 2 shows the results of line patterning at several scan rates for both the CNF probe (Figure 2a) and the Si probe (Figure 2b). Eight different writing speeds have been tested, ranging from 0.5 μm/s to 4 μm/s with increments of 0.5 μm/s. The common bias voltage is 20 V. An apparent correlation of line height and width with the writing speed is observed; the slower the scan rate the higher and wider the patterned line. This phenomenon is expected as a result of longer reaction times [20], and clearly applies for both the CNF and the bare Si probe. For this writing speed series, line height ranges are, respectively, 0.7–2 nm and 0.2–1.2 nm for the CNF and Si probe. The lines show a very good uniformity.

**Figure 3:**
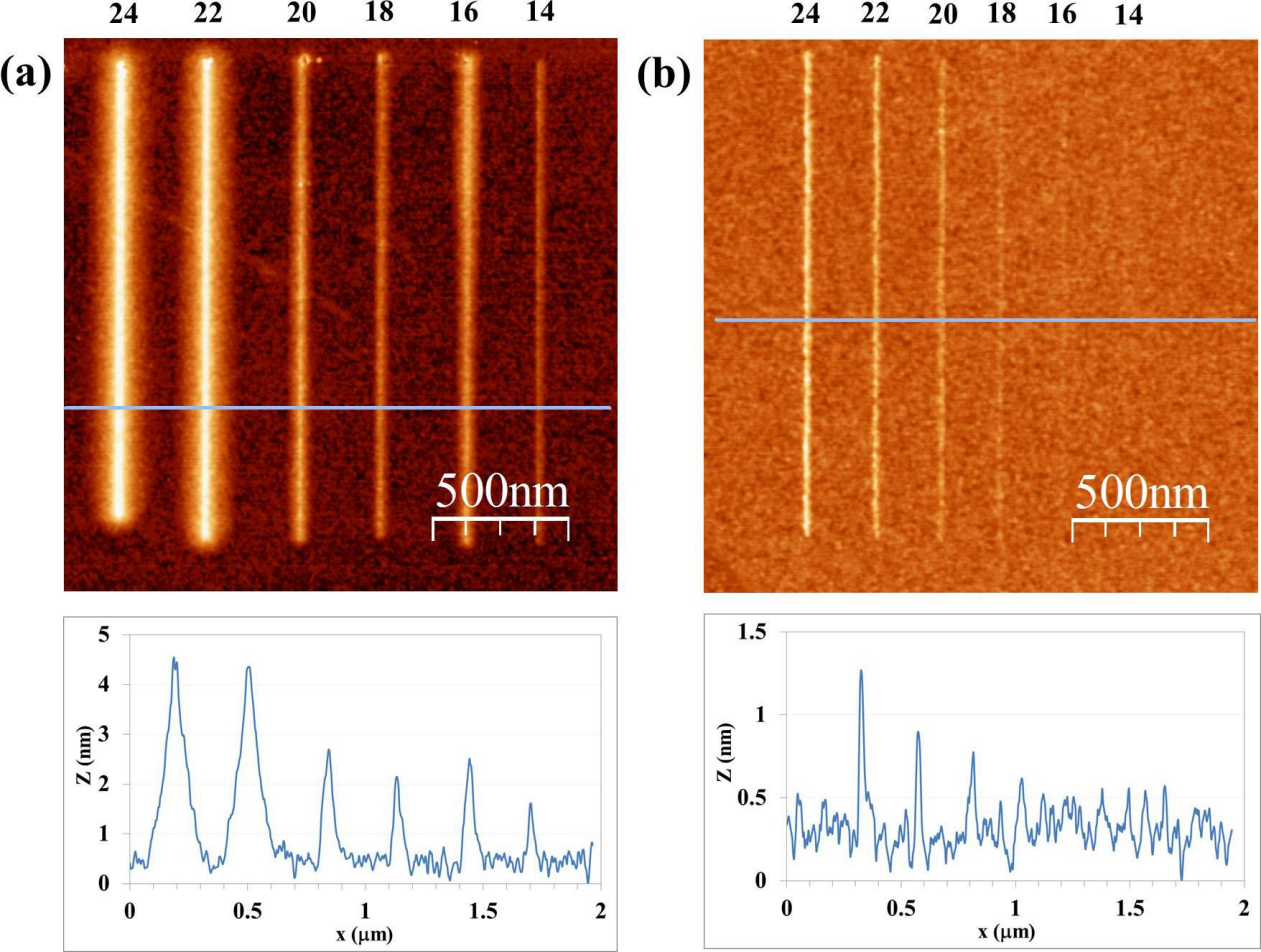
Results of LAO-AFM on Si with CNF (a) and Si (b) probes as a function of the bias voltage (in V). Writing speed is 1 μm/s, RH is 36%.

A similar comparison test of a CNF probe and a Si probe is performed to evaluate the dependence of LAO-AFM line patterns upon the applied bias voltage. The results are shown in Figure 3. Six different bias voltages have been tested, ranging from 14 V to 24 V with increments of 2 V. Common writing speed is 1 μm/s. Again, an apparent correlation of line height and width with bias voltage is obtained; the higher the bias voltage the higher and wider the line pattern. This phenomenon is understood as an indication of the role of the electric field in the kinetics the oxidation [20], which, similar to the results in Figure 2, it is found for both the CNF (Figure 3a) and the bare silicon probe (Figure 3b). For this series of bias voltages, the obtained line height ranges are, respectively, 1.3–4.2 nm and 0.1–1 nm for the CNF and the Si probe.

The definition of line features is good, although some morphological features arise. On the one hand, for Si probe line patterns done at 14 V and 16 V are nearly not quantifiable; their line height and width are in the limits of AFM resolution and Si surface roughness. On the other side, for CNF-patterned lines, an unexpected widening of the line features is especially characteristic of the 22 V and 24 V tests. The analysis of the line profile suggests that the resulting line is a convolution of the typical sharp line features, as obtained, e.g., when using 14 V, with a more delocalized lateral chemical reaction. This aspect would suggest additional mechanisms in addition to the induced main anodic reaction induced by the electric field, such as an ionic diffusive regime of oxidation, which has already been observed [9,21].

### Chemical–mechanical robustness of CNF-AFM probes

**Figure 4:**
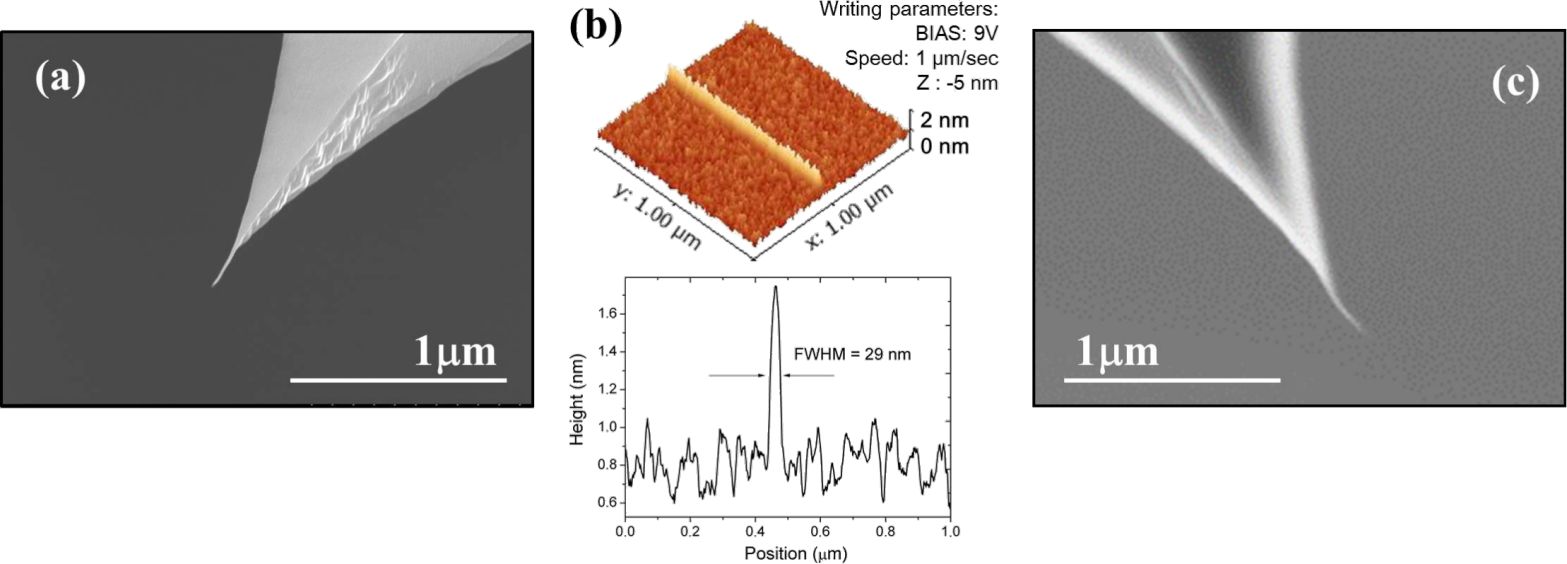
Monitoring chemical and mechanical stability of CNF probes for LAO-AFM. SEM images before (a) and after (c) the definition of 1 μm long line feature, written at 9 V and a writing speed of 1 μm/s (b). The FWHM of the line is about 29 nm.

The chemical and mechanical robustness of CNF has been preliminarily tested. As an example, we monitored the eventual change of the CNF morphology and orientation upon LAO-AFM, as shown in Figure 4. In the left panel (Figure 4a), a SEM image of the CNF-AFM probe before the line patterning by LAO-AFM depicted in Figure 4b is shown. It is compared against a SEM image of CNF-AFM probe after LAO-AFM in the right panel (Figure 4c). Morphological changes of the CNF could not be noticed by SEM inspection. Images using a larger magnification have not been performed in order to avoid contamination and eventual electron-beam-induced damage on the CNFs. These simple characterization results support the suitability of using CNF for LAO-AFM in the dynamic mode of operation. It should be noted that this occurs in spite of the relative weakness of the mechanical clamping of the CNF onto the Si apex. The bending elasticity of the CNF-Si probe upon mechanical contact above a few nanonewtons can be compromised to permanent bending (buckling), particularly for longer CNFs, or rupture [22]. Nevertheless, CNF-probes can be ordinarily used for dynamic mode imaging.

### Reliability of CNF-LAO-AFM

**Figure 5:**
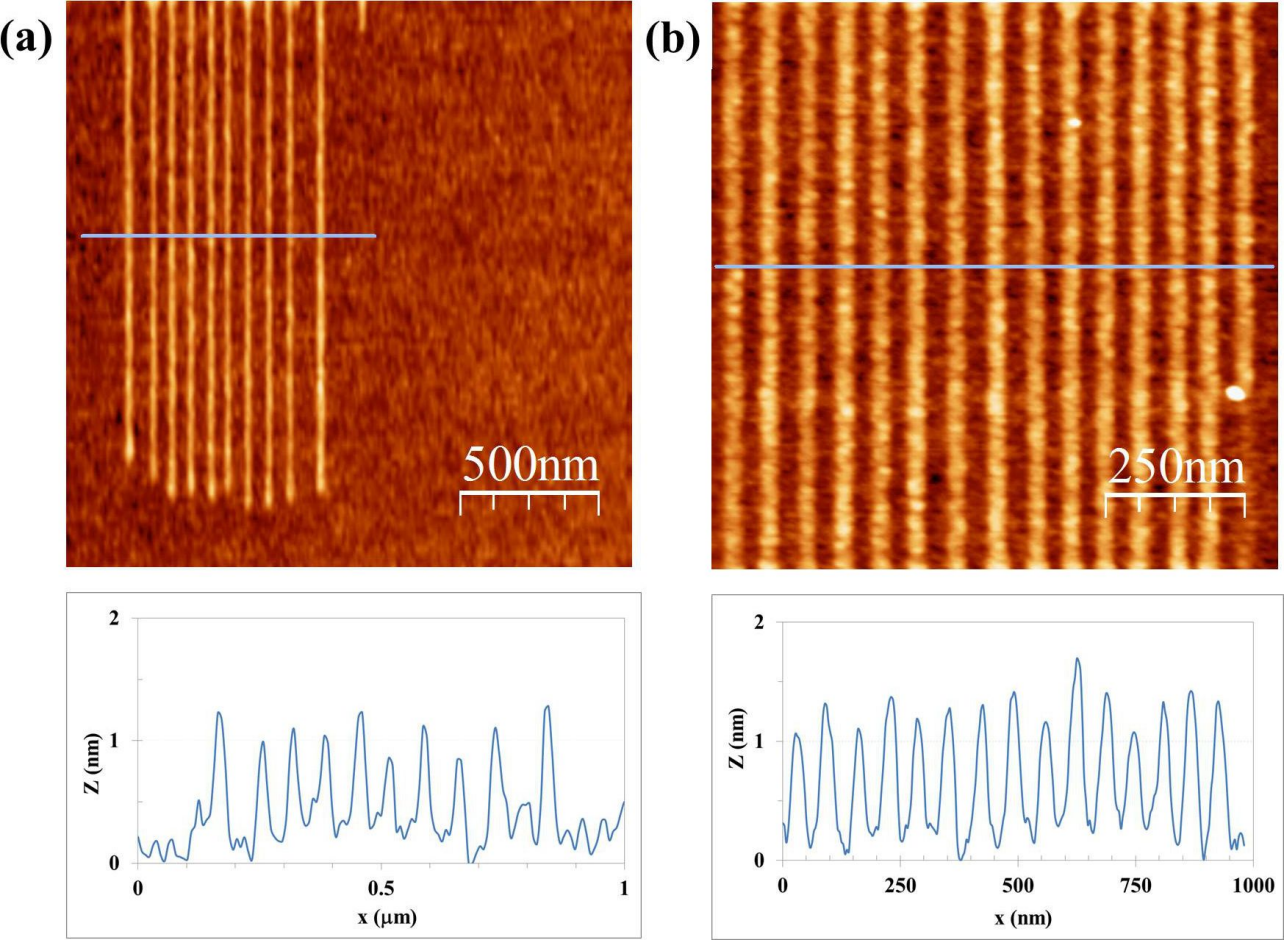
Reproducibility for patterning line arrays by using CNF probes. Array of lines written at a) 23.4 V and b) 43.2 V, having line height of ca. 0.8 nm and ca. 1.2 nm, respectively. The common writing speed is 4 μm/s, RH is 30%.

In Figure 5 we demonstrate another aspect concerning the reliability of CNF probes for LAO-AFM. The images depict two arrays of relatively dense lines defined at two different voltages and at a writing speed of 4 μm/s. Figure 5a corresponds to ten lines defined at 23.4 V, and Figure 5b displays fifteen lines written at 43.2 V. Both voltages were sustained by the CNF and the reproducibility of the line patterning is worth mentioning, because the line variations are well within the intrinsic tolerance of LAO-AFM on Si. Limitations of CNF for pattern density, as well as, for example, chemical inertness upon even stronger electric field should be further investigated by means of dedicated experiments.

Imaging and oxidation is performed in dynamic mode, i.e., under avoidance of tip–surface contact. This is a key aspect of present paper. As far as the tips did not make contact with the surface (either by particle contamination or the surface or problems with feedback loop control) we did not observe tip wear.

## Discussion

**Figure 6:**
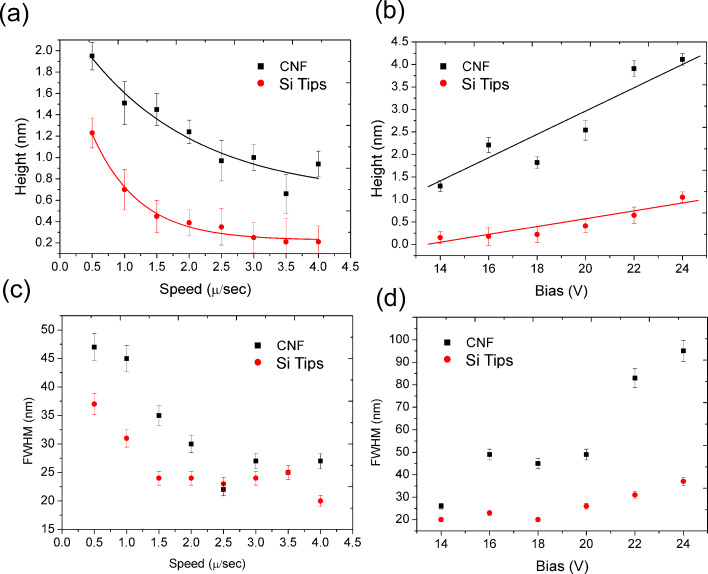
Kinetics of LAO-AFM on Si by using CNF versus Si probes. a) Line height upon writing speed for a bias voltage of 20 V. b) Line height upon bias voltage for a writing speed of 1 μm/s. Full width at half maximum (FWHM) of the same features upon writing speed (c) and upon bias voltage (d).

In Figure 6 the main results of the kinetics study of LAO-AFM are summarized. Figure 6a shows the line height of SiO*_x_* as a function of the writing speed, patterned at a bias voltage of 20 V. The height was determined by averaging ten scan lines. As mentioned above, the oxide growth rate depends inversely upon the writing speed, for both kinds of probes. However, the oxide growth rate by using a CNF probe is significantly higher than that of the bare-Si probe. The proposed exponential decay fit for the experimental data is shown in Figure 6a. The growth rate, expressed here as the oxide height at a certain writing speed, is almost double for the CNF probe.

The linear dependence of oxide line height upon voltage is shown in Figure 6b. Even if there is a higher dispersion for the CNF probes data that could undermine the proposed linear fitting, the linearity of voltage dependence during the LAO process is strongly supported by the literature for Si tips [3,19,23–25]. What limits the thickness of grown SiO*_x_* is not only the decrease of the strength of the electric field as the SiO*_x_* becomes thicker, but also other self-limiting mechanism which decrease the permeability of the hydroxy anions at a given electric field, such as charge build-up in the oxide [21]. Furthermore, one concern when the bias voltage is increased to speed up the oxidation process is the integrity of the tip. Metal-coated probes, as well as Si probes, experience material deposition/sputtering and breakdown voltage at higher bias voltages, like 50 V. We plan to challenge CNF probes in future experiments. In any case, it is evident that the oxidation rate is enhanced when using CNF probes for a given bias voltage. In Figure 6c and Figure 6d we report full width at half maximum (FWHM) values of the same line features mentioned above. Concerning the exposure time (speed) no great change has been observed when comparing CNF against Si tips. However, in the tested voltage range we could observe a strong widening of the features when the voltage exceeds 14 V. This is probably due to a different wettability of the CNF tips which results in a wider water neck when the voltage exceeds a certain critical value.

Additional features arise from the comparison between CNF probe and Si probe performance for LAO-AFM results. We have observed that high resolution (sub 20 nm line width) patterns are much easily obtainable by using CNF probes. As the water meniscus mediates the oxidation reaction kinetics, we hypothesize that the chemical properties of the CNF benefit the generation of a narrower water meniscus, while at the same time maintaining a higher oxidation rate. In this sense, the limited electrical conduction of CNF is not a restrictive point, and the interplay between chemical and electrical properties of the tip material combines to better regulate the oxidation process. Clearly correlated behaviors have been investigated and confirmed for CNT-based LAO-AFM [26–27].

## Conclusion

CNF-AFM probes have been tested for the first time as a tool for nanopatterning based on LAO-AFM in the amplitude modulation dynamic mode. CNF-AFM probes provide suitable electrical, mechanical and chemical properties as required under the experimental conditions of LAO-AFM. We have found experimental evidence that CNF-functionalized Si-probes outperform bare-Si probes for LAO-AFM on a Si surface. Remarkably, CNF-based LAO-AFM shows an increased oxide growth rate, compared to bare-Si probes which we attribute to the shape and chemistry of the CNF tip. Particularly, concentration of the electric field due to the high aspect ratio provided by the CNF apex and changes in wettability, affecting water meniscus shape, with respect to bare Si tip apex are the two mechanisms that would explain the boost in efficiency of CNF for LAO-AFM on silicon. The combination of an increased oxidation rate and an improvement in patterning resolution provided by the geometry of the CNF makes CNF-AFM probes very promising for further developments of LAO-AFM. Future works will fundamentally address the unlocking of CNF-based LAO-AFM variables.
